# Population pharmacokinetics and dosing optimization of unbound teicoplanin in Chinese adult patients

**DOI:** 10.3389/fphar.2022.1045895

**Published:** 2022-11-23

**Authors:** Wen-Qian Fu, Ting-Ting Tian, Min-Xin Zhang, Hong-Tao Song, Li-Li Zhang

**Affiliations:** ^1^ Department of Pharmacy, 900th Hospital of Joint Logistics Support Force, Fuzhou, China; ^2^ Department of Purchasing Management, 900th Hospital of Joint Logistics Support Force, Fuzhou, China

**Keywords:** unbound teicoplanin, Chinese adult patients, population pharmacokinetics, dosing optimization, Monte Carlo simulation

## Abstract

**Objectives:** To develop a population pharmacokinetic (PopPK) model describing unbound teicoplanin concentrations in Chinese adult patients and perform Monte Carlo simulations to optimize the dosing regimens.

**Methods:** The raw data for PopPK analysis in this study were collected from Chinese adult patients. A PopPK model of unbound teicoplanin was developed and Monte Carlo simulations were used to optimize the dosing regimens. The trough concentrations of unbound teicoplanin were targeted at 0.75 mg/L and 1.13 mg/L for most infection induced by Gram-positive bacteria and endocarditis or severe infections, respectively.

**Results:** A total of 103 teicoplanin unbound concentrations were collected from 72 Chinese adult patients. A one-compartment pharmacokinetic model with first-order elimination was established. The typical values of clearance and the volume of distribution were 11.7 L/h and 811 L, respectively. The clearance and volume of distribution of unbound teicoplanin were positively correlated with estimated glomerular filtration rate (eGFR) and serum albumin concentrations, respectively. Dosing simulation results showed that standard dosing regimens were unable to meet the treatment needs of all patients, and the dosing regimen need optimize based on eGFR and serum albumin concentrations. The high eGFR and serum albumin concentration were associated with reduced probability of achieving target unbound trough concentrations.

**Conclusion:** We successfully characterized the pharmacokinetics of unbound teicoplanin in Chinese adult patients. Importantly, we further highlight the importance of guiding dosing through unbound drugs. To achieve safe and effective treatment, the dosing regimens need to be adjusted according to eGFR and serum albumin concentrations.

## Introduction

Teicoplanin is a glycopeptide antibiotic and is widely used in the treatment of serious infections caused by drug-resistant Gram-positive bacteria, such as methicillin-resistant *Staphylococcus aureus* (MRSA), methicillin-resistant coagulase-negative *Staphylococci* and penicillin-resistant *Streptococcus pneumonia* (Lu and Song 2014). A review and meta-analysis that included 24 randomized controlled trials concluded that teicoplanin is not inferior to vancomycin with regard to efficacy and is associated with lower adverse events rate than vancomycin (Svetitsky et al., 2009).

Teicoplanin has time-dependent antibacterial activity with evident post-antibiotic effects and has a long half-life of elimination (30–180 h) (Li and Wang, 2016; Gao et al., 2020). Teicoplanin clinical efficacy is closely associated with trough concentration (C_trough_). For most infection induced by Gram-positive bacteria, the suggested therapeutic total C_trough_ is no less than 10 mg/L (detected by HPLC method); For endocarditis and severe infection, the suggested therapeutic total C_trough_ is no less than 15 mg/L (detected by HPLC method). Previous studies have shown that adverse events increased significantly when C_trough_ exceeds suggested range. Thrombocytopenia was more common at total C_trough_ > 60 mg/L (Pauluzzi et al., 1987), so the therapeutic total C_trough_ is suggested to not exceed 60 mg/L (Tobin et al., 2010). At present, total C_trough_ is used to guide dosing, however, the standard dosing regimens (three loading doses of 400 mg q12h followed by maintenance doses of 400 mg/200 mg qd) may not consistently achieve the therapeutic concentrations.

Importantly, teicoplanin is highly bound to serum albumin (90%–95%) (Gao et al., 2020) and the majority of drug is excreted unchanged in the urine by glomerular filtration (Rowland, 1990; Tobin et al., 2010). Only the unbound (free) teicoplanin is able to distribute into body tissues and exert pharmacological (antibacterial) activity. Previous studies demonstrated the level of serum albumin was an important determinant of teicoplanin pharmacokinetic (PK) variability. Hypoproteinemia (serum albumin < 25 g/L) could result in higher unbound fractions and large variability of protein binding ratio (Yano et al., 2007; Roberts et al., 2014; Byrne et al., 2017a). Theoretically, one can expect higher active concentrations in patients with hypoproteinemia. However, increased unbound fractions may result in increased distribution and clearance (because glomeruli only filtrate the unbound drug), which could reduce total concentrations (Ulldemolins et al., 2011). Therefore, it might be not comprehensive to evaluate the efficacy and safety of teicoplanin only by total concentrations (Aulin et al., 2021). Meanwhile, for the antibacterial drugs whose efficacy are evaluated by trough concentrations, the correlation between unbound concentrations and efficacy is greater than that of total concentrations (Brink et al., 2015). Consequently, in the clinical, while monitoring the total concentrations, it is also necessary to focus on the unbound concentrations of the patients.

According to the estimation method in previous study (Byrne et al., 2018), the therapeutic unbound teicoplanin C_trough_ was calculated based on the suggested therapeutic total C_trough_ described above and the protein binding ratio (90%–95%). In this study, the protein binding ratio was calculated as the average value of 92.5%. For the most infection induced by Gram-positive bacteria, the suggested therapeutic unbound C_trough_ was no less than 0.75 mg/L. For endocarditis and severe infection, the suggested therapeutic unbound C_trough_ was no less than 1.13 mg/L. And the unbound C_trough_ was suggested to not exceed 4.5 mg/L.

Recently, Therapeutic drug monitoring (TDM) combined with population pharmacokinetics (PopPK) are commonly used to achieve individual dosing (Su et al., 2015). Since the 1990s, there have been several teicoplanin PopPK studies (Ramos-Martín et al., 2014; Byrne et al., 2017b; Kasai et al., 2018), but most studies to date were based on foreign population, and focused on total teicoplanin concentrations rather than unbound concentrations. Considering the ethnic differences, it might not be appropriate to directly extrapolate the PopPK model to the Chinese population. To ensure the safety and efficacy of teicoplanin in Chinese adult patients, this study collected unbound teicoplanin concentrations and other clinical data of Chinese adult patients, developed a PopPK model describing unbound teicoplanin and performed Monte Carlo simulations to propose optimal dosing regimens likely to achieve suggested therapeutic unbound concentrations in Chinese adult patients.

## Materials and methods

### Study population and data collection

This was a prospective study, the raw data of teicoplanin used for the PopPK analysis were collected from patients treated with teicoplanin at *900th Hospital of Joint Logistics Support Force* between January 2019 and December 2019. Patients aged 18 years or older treated with teicoplanin intravenously were included. The pregnant female patients, hemodialysis patients, disseminated intravascular coagulation patients, and continuous renal replacement therapy patients were excluded.

Data used for PopPK analysis in this study included, but were not limited to, demographics (gender, age, and weight [WT]), physiological and biochemical parameters (serum creatinine [Scr], blood urea nitrogen [BUN], cystatin C [Cys C], white blood cell [WBC] and serum albumin), dosing information (trade name, dose, infusion time, administration rate, administration interval), and PK sampling information (sampling time, unbound concentrations), etc. Estimated glomerular filtration rate (eGFR) was estimated by CKD-EPI equations (Center for Drug Evaluation, 2021):

$$eGFR=144\times {\left(\frac{Scr}{0.7}\right)}^{-0.329}\times 0.993^{Age},if\;female\;and\;Scr\leq 0.7$$ (1)

$$eGFR=144\times {\left(\frac{Scr}{0.7}\right)}^{-1.209}\times 0.993^{Age},\;if\;female\;and\;Scr>0.7$$ (2)

$$eGFR=141\times {\left(\frac{Scr}{0.9}\right)}^{-0.411}\times 0.993^{Age},\;if\;male\;and\;Scr\leq 0.9$$ (3)

$$eGFR=141\times {\left(\frac{Scr}{0.9}\right)}^{-1.209}\times 0.993^{Age},\;if\;male\;and\;Scr>0.9$$ (4)

Note: Age: years, Scr: mg·dL^−1^, 1 mg dL^−1^ = 88.4 μmol L^−1^

This study has been approved by the Ethics Committee of *900th Hospital of Joint Logistics Support Force*, and all subjects signed informed consent form.

### Teicoplanin dosing, blood sampling, and measurement

Teicoplanin was administered intravenously by infusion for 40 min. According to the relevant guidelines (Li and Wang, 2016), the standard dosing regimens of teicoplanin was 400 mg every 12 h for three doses followed by 400 mg/200 mg once daily. However, prescribed dosing regimens were at the discretion of treating physicians based on the severity of the patient’s disease and the standard dosing regimen was not always followed. The daily dose range of patients included in this study was 50–1,600 mg.

Blood samples were typically collected within 30 min to 1 h preceding the 4th dose and the 6th dose, and depending on the actual clinical situation, blood samples might be taken at other time for TDM during the treatment. The Plasma samples were placed in a Centrifree^®^ ultrafiltration device and placed in a 37°C water bath for 30 min, followed by centrifugation at 37°C, 1,500 × *g* for 30 min, and the ultrafiltrate was directly used for the determination of unbound concentration by ultra-performance liquid chromatography-tandem mass spectrometry (UPLC-MS/MS) method. The calibration curve of unbound teicoplanin in plasma was linear over the range of 0.10–8.00 μg/ml (*r* = 0.999). The intra-assay precision and the inter-assay precision of samples did not exceed 7.00%. The average relative recovery ratio was 97.9%, and the matrix effect factor was 0.97. Details of blood handling, storage and measurement have been described previously (Fu et al., 2020).

### Population pharmacokinetic analysis

The PopPK modeling was performed using NONMEM (non-linear mixed effects modeling, v7.2, Globomax Corp, United States), and Wings for NONMEM (v6.1, Nick Holford, University of Auckland, New Zealand) was used as an auxiliary software for NONMEM execution. R (v3.6.1, Saik Urien, U.R.C. Paris Centre, Hopital Tarnier, France) was used for exploratory data analysis, data assembly, and graphical presentation of modeling and simulation results.

One-compartment (ADVAN1 TRANS2) and two-compartment (ADVAN3 TRANS4) PK models with first-order elimination were used to fit the PK observations of unbound teicoplanin. The parameter estimation method for PopPK model was FOCEI (first order conditional estimation with η-ε interaction option). The inter-individual variability (IIV) of the parameters were estimated using exponential random effects.

$$\theta_{i}=\theta ∙e^{\eta_{i}}$$ (5)

Where θ_i_ is the estimated parameter value of the individual i, θ represents the typical population parameter, and η_i_ (IIV) is assumed to follow the normal distribution with mean of 0 and variance of ω^2^ and was estimated during model fitting.

The additive, proportional, and mixed residual error models were compared separately, and finally the mixed residual error model was selected for data fitting.

$$Y=F\times \left(1+\varepsilon_{1}\right)+\varepsilon_{2}$$ (6)

where *Y* represents the observed unbound teicoplanin concentrations, *F* is the individual prediction, and *ε*_1_ and *ε*_2_ are the proportional error and additive error, respectively, which followed the normal distributions with mean of 0 and variance of *σ*_1_^2^ and *σ*_2_^2^, respectively.

### Covariate analysis

Covariate modeling analysis was used to explore the source of variability. Age, gender, WT, Scr, BUN, Cys C, serum albumin, eGFR, WBC and the teicoplanin type (teicoplanin produced by Sanofi-Aventis S. p.A or HISUN) received during teicoplanin therapy were investigated as potential variables on PK parameters.

The stepwise covariate modeling approach was used to establish the covariate model in this analysis. During the forward selection step, all covariates were added to the PK parameters in the base model one by one. Covariates were screened based on the changes in objective function values (OFV) and graphical evaluation. The covariate with a significant effect (*p* < 0.05, e.g., df = 1, ΔOFV ≥ 3.84) would be added to the basic model to build a full model. Based on the full model, the existing covariates were deleted one by one as the backward elimination step. For each deleted covariate, the increase in OFV should be ≥ 10.83 (*p* < 0.001, df = 1), otherwise, this covariate should be excluded from the final model.

Continuous covariates (e.g., WT) were added to the model according to the following equation.

$$\theta_{i}=\theta_{TV}\times {\left(\frac{cov_{i}}{cov_{median}}\right)}^{\theta_{x}}$$ (7)

Where θ_i_ is the parameter value of the *i*th individual; θ_TV_ is the population typical value of PK parameters; cov_i_ is the covariate value of the *i*th individual; cov_median_ is the median value of this covariate; θ_x_ is the influence coefficient of the covariate on the parameters.

Categorical covariates (e.g., gender) were added to the model according to the following equations:

$$\theta_{i}=\theta_{TV}+\theta_{x}{,}_{cov=x_{l}}\;if\;Cov=X_{l}$$ (8)

$$\theta_{i}=\theta_{TV}\;if\;Cov=X_{0}$$ (9)

Where θ_x,cov=xl_ is the change in a parameter when covariate X is at level l compared with the reference level (0).

### Model evaluation

To evaluate the accuracy, appropriateness and stability of the final model, goodness-of-fit plots, non-parametric bootstrap, normalized prediction distribution errors (NPDE) were performed.

Goodness-of-fit plots were used to assess the appropriateness of the model. It included the plots of observed values against individual or population predictions and conditional weighted residuals (CWRESs) against time or population predictions.

The performance and stability of the final model were estimated using non-parametric bootstrap. 1,000 datasets generated by random sampling were evaluated. The 95% confidence interval and median of the final parameters were calculated and compared to the final parameters estimated by NONMEM program.

The descriptive performance of the model was evaluated by calculation of the NPDE. 1,000 times simulations were performed based on the final model. The results were used for plotting, including quantile-quantile plot, the NPDE histogram, NPDE versus time and PRED plots.

### Simulation and dosing optimization

Monte Carlo simulations were performed for adult patients (*n* = 1,000) based on the final PopPK model. Six levels of eGFR (20, 30, 45, 60, 90, and 130 ml/min) and six levels of serum albumin concentrations (15, 20, 25, 30, 35, and 40 g/L) were evaluated. Three loading doses (q12h) followed by maintenance doses (qd) were simulated. The probability of target attainment (PTA) for achieving a target unbound C_through_ of 0.75 mg/L (most infection induced by Gram-positive bacteria) or 1.13 mg/L (endocarditis and severe infection) at 48 h and 96 h was calculated. The PTA (risk) of achieving the unbound C_through_ of 4.5 mg/L at 96 h (C_through_ ≥ 4.5 mg/L means more adverse events)was also calculated. The optimal dosing regimens were finally selected based on above PTA calculations.

## Results

### Study population

Data for the PopPK analysis were obtained from 72 subjects, including 26 female patients and 46 male patients. A total of 103 PK observations were obtained. Demographic information and physiological or biochemical parameters for this analysis are presented in Table 1.

**TABLE 1:** Characteristics of patients included in the study.

Parameters (unit)	Value^a^
Number of patients (male/female)	72 (46/26)
Number of PK observations	103
Age (years)	69 ± 20 (18–99)
WT (kg)	61.0 ± 9.8 (40–85)
Type of teicoplanin/(teicoplanin produced by Sanofi-Aventis or HISUN)	44/28
Daily dose (mg)	50–1,600
Unbound teicoplanin concentrations (μg/ml)	1.5 ± 0.9 (0.4–4.4)
Scr (μmol/L)	86.2 ± 48.3 (32.1–263.0)
BUN (mmol/L)	9.5 ± 7.4 (1.6–36.8)
Cys C (mg/L)	1.4 ± 0.8 (0.6–3.7)
eGFR (ml/min)	80.8 ± 27.1 (17.4–134.0)
Albumin (g/L)	31.0 ± 5.1 (18.3–46.0)
WBC (10^9^/L)	10.6 ± 5.3 (2.1–32.5)
The infection type (%)	
Pulmonary infection	58 (80.6)
Bacteremia	3 (4.2)
Pyemia	4 (5.6)
Abdominal infection	2 (2.8)
Skin infection	6 (8.3)
Hepatapostema	1 (1.4)
Biliary tract infection	2 (2.8)

Note: BUN, blood urea nitrogen; Cys C, cystatin C; eGFR, estimated glomerular filtration rate; PK, pharmacokinetic; Scr, serum creatinine; WBC, white blood cell; WT, weight.

a values are expressed as mean ± SD (range) or as n.

### Population pharmacokinetics modeling

In this analysis, one-compartment and two-compartment models were used as structural models to fit the PK observations. The results showed that one-compartment model could successfully minimize and the parameters were estimated reasonably (OFV = −24.4, condition number = 18.8). Compared with one-compartment model, the two-compartment model showed no significant change in OFV value (ΔOFV = −0.24), and the condition number was large (condition number = 1.16e + 007), at the same time, the IIV of the clearance between peripheral and central compartments and the volume of distribution in peripheral compartment could not be accurately estimated. Based on above analyses, the one-compartment PopPK model with first-order elimination was selected as the basic structural model describing the PK characteristics of unbound teicoplanin.

The results of covariate analysis showed that eGFR was a significant covariate of the clearance (CL) of the unbound teicoplanin, serum albumin concentrations significantly affected the volume of distribution (V) of the unbound teicoplanin, and the model OFV values decreased by 11.037 and 13.442, respectively. Type of teicoplanin, gender, age, WT, Scr, BUN, WBC and Cys C were not significant covariates of the PK parameters of the unbound teicoplanin. The final model could be described using following equations:

$$CL\left(L/h\right)=11.7\times {\left(\frac{eGFR}{84}\right)}^{0.476}\times e^{\eta CL}$$ (10)

$$V\left(L\right)=811\times {\left(\frac{ALB}{32}\right)}^{1.6}\times e^{\eta V}$$ (11)

where CL is clearance, eGFR is estimated glomerular filtration rate, ALB is serum albumin concentrations, V is the volume of distribution. The final model parameter estimates are shown in Table 2.

**TABLE 2:** PK parameter estimates and results of bootstrap analysis for the final model.

Parameter	NONMEM			Bootstrap		Bias (%)
	Estimates	RSE (%)	Shrinkage (%)	Median	95% CI	
θ_CL_ (L/h)	11.7	7.4	—	11.38	9.06–13.4	−2.72
θ_V_ (L)	811	11.1	—	822	616–1,025	1.42
θ_ALB_ (g/L)	1.60	27.4	—	1.55	0.690–2.55	−3.11
θ_eGFR_ (ml/min)	0.476	50	—	0.501	0.229–1.40	5.21
η_CL_ (%)	38.6	16.5	35	36.2	17.9–49	−6.17
η_V_ (%)	53.7	13.2	31	51.9	23.6–71.0	−3.34
ε_1_ (%)	18.3	14.5	36	17.8	9.04–25.0	−3.03
ε_2_ (μg/ml)	0.122	41.3	36	0.119	0.0207–0.198	−2.33

Note: θ_CL_, The population parameters typical value of clearance; θ_V_, The population parameters typical value of the volume of distribution; θ_ALB_, The population parameters typical value of ALB; θ_eGFR_, The population parameters typical value of eGFR; η_CL_, Inter-individual variation in clearance; η_V_, Inter-individual variation in the volume of distribution; ε, Residual variation; CI, Confidence interval; RSE, relative standard error; Bias, prediction error; Bias% = (Bootstrap Median–NONMEM Estimates)/NONMEM Estimates × 100%.

### Model evaluation

**FIGURE 1:**
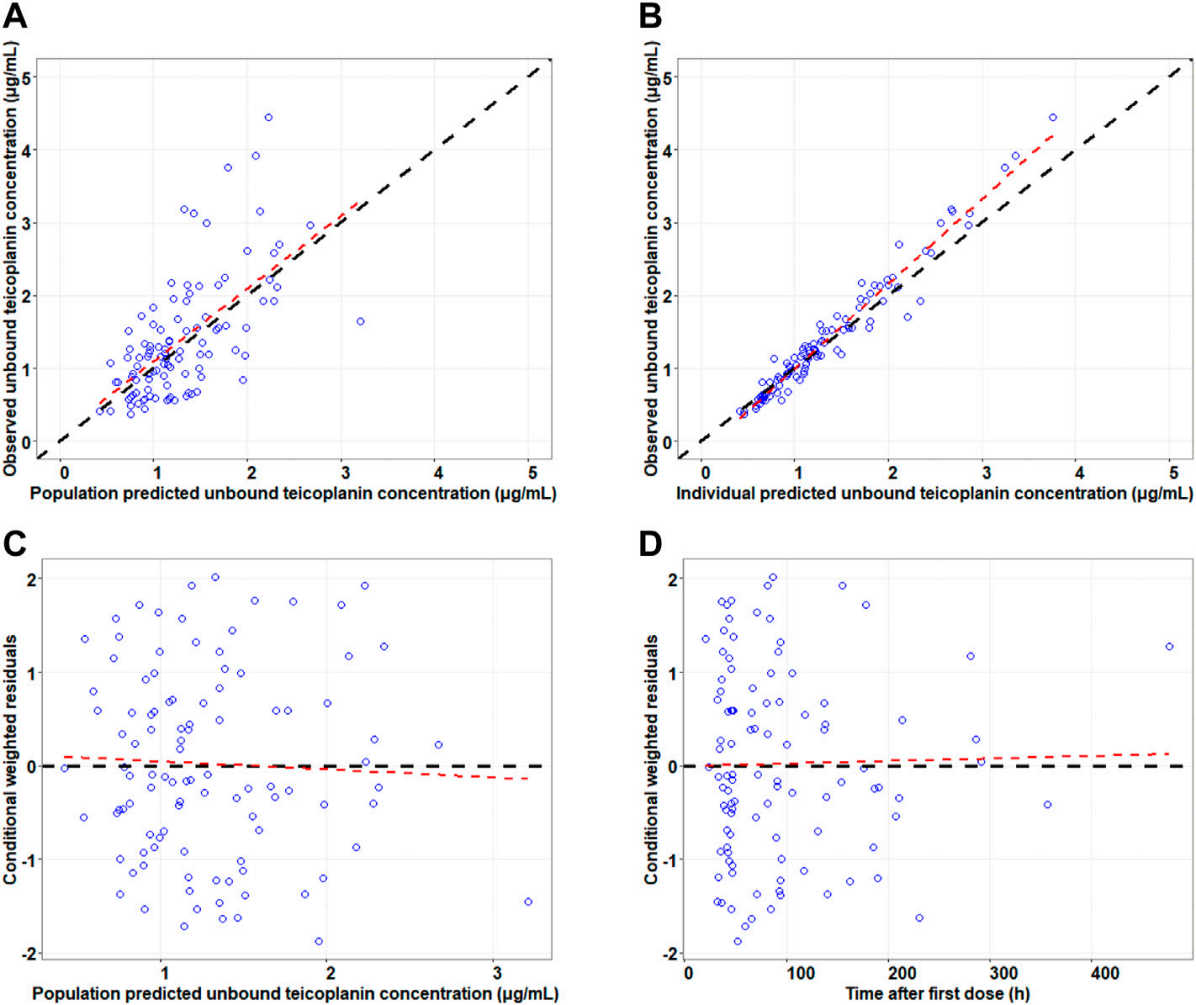
Goodness-of-fit plots for the final model. Note: The upper left and upper right panels represent observed versus population and individual predicted concentrations, respectively **(A,B)**; the lower left and lower right panels represent conditional weighted residuals versus population predicted concentrations and the time after the first dose, respectively **(C,D)**. The line in the upper panel is y = x; the line in the lower panel is y = 0.

The goodness-of-fit plots for final model are presented in Figure 1. The results of model diagnosis showed that population predicted versus observed concentrations were evenly distributed around the line of y = x, and less scatter points deviated far from the line of y = x. The individual predicted versus observed concentrations were evenly distributed around the line of y = x. Conditional weighted residuals versus population predictions and time after the first dose were evenly distributed around the line of y = 0. The model diagnostic plots showed that the established model fit the data well, and the selected error model was adequate.

The parameter estimates of the final model and the results of non-parametric bootstrap analysis are shown in Table 2. The bootstrap results showed the successful minimization in 875 of the 1,000 simulations. The median values were close to the final parameters estimated by NONMEM program, with <6.5% bias, and the final model parameter estimates were within the 95% confidence intervals of bootstrap results, indicating that the performance of the model was stable.

**FIGURE 2:**
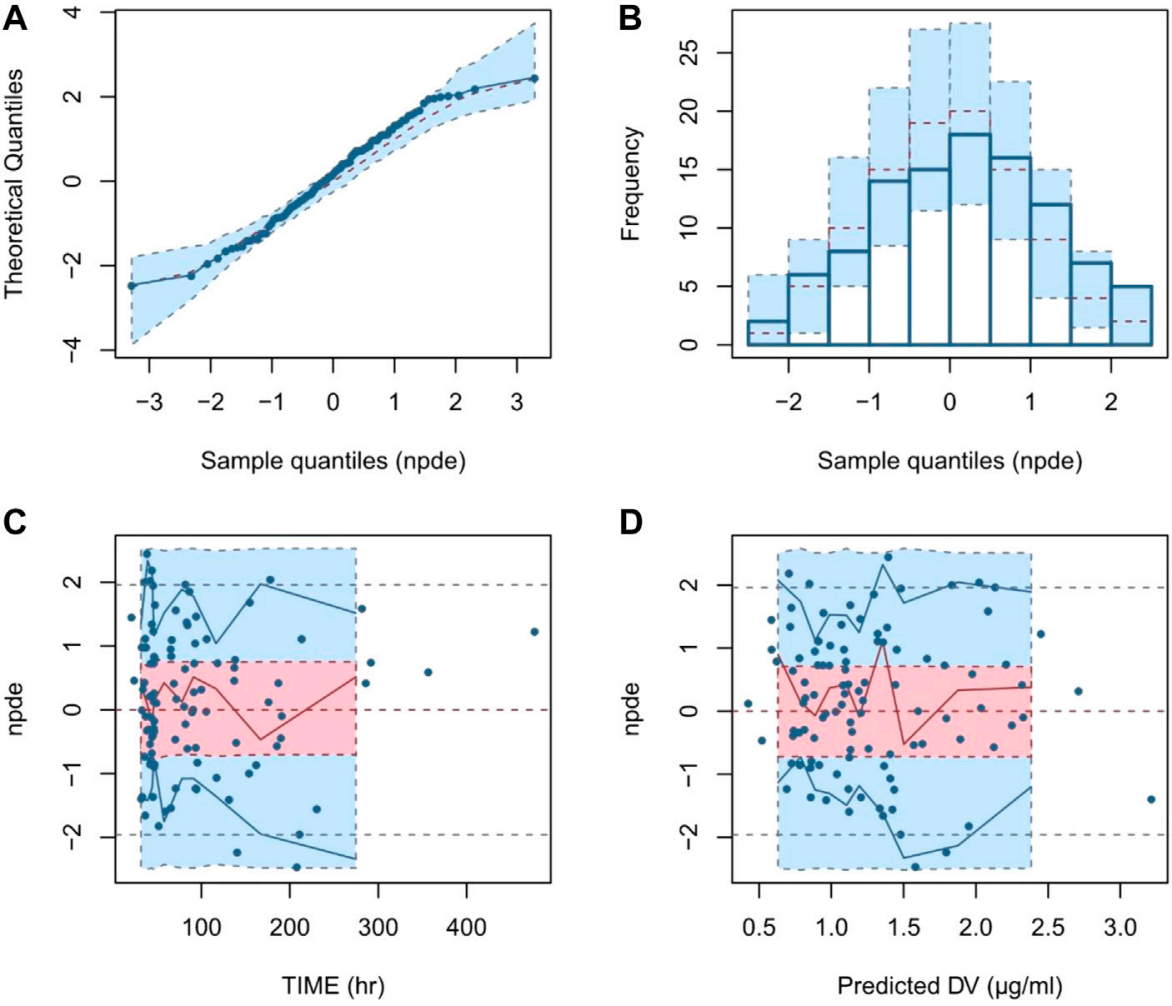
Normalized prediction distribution error (NPDE) plots of final model. Note: **(A)** quantile-quantile plot of the distribution of the NPDE against theoretical distribution; **(B)** histogram of the distribution of the NPDE against theoretical distribution; **(C)** scatter plot of NPDE vs. time after the first dose (TAFD); **(D)** scatter plot of NPDE vs. predicted concentrations.

Evaluation of the NPDE distribution showed that the mean of the NPDE was not significantly different from 0 (Wilcoxon signed rank, *p* = 0.131 > 0.05), the variance was not significantly different from 1 (Fisher test, *p* = 0.142 > 0.05) and the NPDE distribution was not significantly different from a normal distribution (Shapiro-Wilks, *p* = 0.561 > 0.05). The NPDE plots of the final model are presented in Figure 2. It can be seen that the NPDE followed a normal distribution and no trend in the scatterplots was observed. The results confirmed that the final model could adequately describe the observed data.

### Simulation and dosing optimization

**FIGURE 3:**
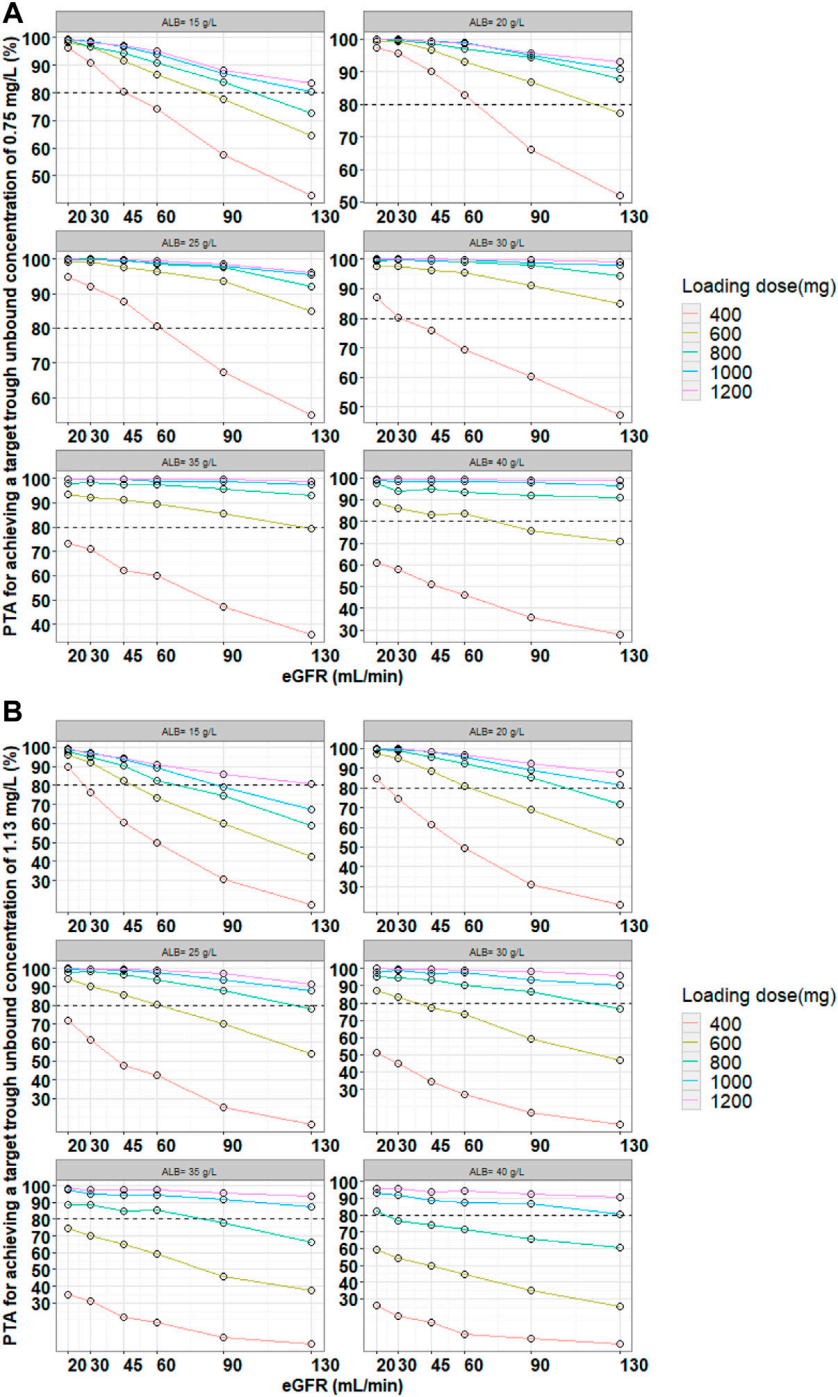
Monte Carlo simulations and PTA for unbound trough concentrations at 48 h for various loading dose regimens. Note: Loading doses were administered every 12 h for three doses and unbound trough concentrations were simulated at 48 h. The dash line represented a PTA of 80%. **(A)** Most infection induced by Gram-positive bacteria, **(B)** endocarditis and severe infection.

Monte Carlo simulations were performed based on the final model and the simulated population was stratified by the various eGFR and serum albumin levels. The PTA for achieving a target unbound C_through_ of 0.75 mg/L (most infection induced by Gram-positive bacteria) or 1.13 mg/L (endocarditis and severe infection) at 48 h or 96 h were calculated. The PTA results for the various teicoplanin loading dose regimen simulations for unbound C_through_ at 48 h are shown in Figure 3. The simulation results showed that PTA decreased as eGFR and serum albumin concentration increased, and PTA increased as the loading dose increased. The standard loading dose regimen of 400 mg for three doses could only achieve PTA ≥ 80% in patients with low eGFR or serum albumin concentrations, and higher loading doses were required in patients with high eGFR and serum albumin concentrations. In addition, with the same eGFR and serum albumin concentrations, patients with endocarditis and severe infection require a higher loading dose to achieve PTA ≥ 80%. Table 3 summarizes the dosing regimens (loading dose and maintenance dose) with ≥ 80% PTA at 48 h or 96 h, and the probability (risk) of achieving unbound C_through_ ≥ 4.5 mg/L at 96 h for each dosing regimen. The simulation results for the recommended dosing regimens are provided as Supplementary Material.

**TABLE 3:** Optimal dosing regimens achieving target unbound teicoplanin C_through_ at 48 h for loading dose regimens and at 96 h for maintenance dose regimens (PTA ≥ 80%).

eGFR (ml/min)	ALB (g/L)					
	15	20	25	30	35	40
Most infection induced by Gram-positive bacteriaLoading dose (mg)/maintenance dose (mg) to achieve unbound target C_through_ of 0.75 mg/L [Probability of unbound C_through_ ≥ 4.5 mg/L at 96 h (%)]						
20	400/400 (1.7)	400/200 (0)	400/200 (0)	400/200 (0)	600/200 (0)	600/200 (0)
30	400/400 (0.4)	400/400 (0)	400/200 (0)	400/200 (0)	600/200 (0)	600/200 (0)
45	400/400 (0.1)	400/400 (0)	400/400 (0)	600/200 (0)	600/200 (0)	600/200 (0)
60	600/600 (0.8)	400/400 (0)	400/400 (0)	600/400 (0)	600/400 (0)	600/400 (0)
90	800/800 (1.7)	600/600 (0)	600/600 (0)	600/400 (0)	600/400 (0)	800/400 (0)
130	1,000/1,000 (1.7)	800/800 (0.4)	600/600 (0)	600/600 (0)	600/600 (0)	800/400 (0)
Endocarditis and severe infectionLoading dose (mg)/maintenance dose (mg) to achieve unbound target C_through_ of 1.13 mg/L (Probability of unbound C_through_≥4.5 mg/L at 96 h [%])						
20	400/400 (1.7)	400/400 (0.5)	600/200 (0.2)	600/200 (0.2)	800/200 (0.3)	800/200 (0.4)
30	600/600 (5.3)	600/400 (0.7)	600/400 (0.1)	600/400 (0.1)	800/400 (0.4)	1,000/200 (0)
45	600/600 (2.6)	600/600 (1.5)	600/600 (0.4)	800/400 (0.4)	800/400 (0)	1,000/400 (0.2)
60	800/800 (4.6)	600/600 (0.7)	800/600 (1.0)	800/600 (0.2)	800/600 (0.4)	1,000/400 (0.1)
90	1,000/1,000 (4.6)	800/800 (1.6)	800/800 (0.9)	800/600 (0.2)	1,000/600 (0.2)	1,000/600 (0.1)
130	1,200/1,200 (4.7)	1,000/1,000 (0.2)	1,000/800 (0.4)	1,000/800 (0)	1,000/800 (0)	1,000/800 (0)

Note: ALB, albumin; C_trough_, trough concentration; eGFR, estimated glomerular filtration rate; PTA, probability of target attainment.

## Discussion

This study was the first to establish a PopPK model of unbound teicoplanin in adult Chinese patients and assess the effect of intrinsic/extrinsic factors on the PK characteristics of unbound teicoplanin. At the same time, the optimal dosing regimens for achieving target unbound C_trough_ were obtained by Monte Carlo simulation, providing a reference for clinical application.

In this study, due to the limitation of clinical circumstances, it was difficult to perform relatively intensive PK sampling to describe the complete concentration-time profiles. The sampling method was sparse sampling, with an average of only 1-5 sampling points per patient, and most were trough concentrations. This was close to the sampling method in the study by Kasai et al. (2018) and Byrne et al. (2018). Such sampling data could accurately describe the elimination phase. A one-compartment model was optimal and adopted for the modeling of the data. The results of covariate analysis showed the eGFR was a significant covariate on CL, and serum albumin was a significant covariate on V.

The typical value of CL was 11.7 L/h, which was close to the value of 7.29 L/h for the unbound teicoplanin in the previous study (Byrne et al., 2018). The typical value of V was 811 L, which was greater than the value of total teicoplanin obtained in our previous PopPK study (83.1 L) (Fu et al., 2021). Because teicoplanin is highly bound to serum albumin (90%–95%), it was justified that only unbound drug could distribute into tissues and was more widely distributed than total drug. The renal function (eGFR) was positive correlation with unbound teicoplanin CL, but the relative standard error of eGFR was relatively large (RSE = 50%). It might be due to the large IIV of patients as well as limited sample size. Considering eGFR could significantly improve the goodness-of-fit of the model and the result was also in keeping with teicoplanin’s elimination characteristics (Wilson, 2000), the final model retained eGFR as a covariate. The finding was consistent with the results obtained in the previous studies (Soy et al., 2006; Matsumoto et al., 2016; Ogami et al., 2020). In addition, the serum albumin concentrations of patients included in this study ranged from 18.3 to 46 g/L, covering a wide range, and included patients with hypoproteinemia (<25 g/L). The covariate analysis also revealed that the serum albumin level was positive correlation with V. The volume of distribution was lower in patients with low serum albumin levels, and the unbound concentrations were higher at certain doses. The finding was consistent with the previous studies (Yano et al., 2007; Zhao et al., 2015) that suggested there were higher unbound teicoplanin concentrations in patients with lower ALB levels. Ulldemolins et al. (2011) also proposed that serum albumin concentrations significantly impacts CL or V in highly protein-bound drugs.

It was found that body weight significantly affected the volume of distribution of teicoplanin in patients with haematological malignancy in the previous studies (Byrne et al., 2017b). However, this study did not find the significant effect of body weight on PK parameters of unbound teicoplanin. The possible reasons are as follows: 1) the majority of patients’ (more than 80%) body weight was centrally distributed between 50 and 70 kg; 2) some patients were long-term bedridden and unable to measure the body weight accurately; 3) small sample size with certain limitations.

Based on the parameters of the final model, the dosing regimens were designed by simulation in patients with different levels of eGFR and serum albumin concentrations to achieve the PTA of no less than 80%. We found that the standard dosing regimen (three loading doses of 400 mg q12h followed by maintenance doses of 400 mg/200 mg qd) did not meet the treatment needs of all patients and higher PTA could only be achieved in patients with low eGFR and ALB levels. In particular, a dose increase may be necessary in patients with enhanced renal function and high serum albumin levels, or in patients with endocarditis and severe infection. Meanwhile, patients with renal impairment and hypoproteinemia at the same dose had a relatively increased risk of adverse events (probability of attaining trough unbound concentrations ≥4.5 mg/L). This suggested that in clinical, it would be useful to measure unbound concentrations, and the dosing regimen needed to be adjusted in time according to the patient’s eGFR and serum albumin concentrations.

Several limitations of this study warrant mention. This study was a single center study and a different result may have been obtained if multiple centers had been studied. The main limitation of this study was the sample size of our data set. Although the sample size could meet the needs of PK modeling, it was necessary to expand the sample size while collecting more blood samples obtained by intensive sampling to further evaluate and optimize the model of unbound teicoplanin.

## Conclusion

In conclusion, in order to optimize teicoplanin therapy in adult Chinese patients, a PopPK model of the unbound teicoplanin concentrations was developed in this study and recommendations for individualized dosing regimens were made by simulation. The effects of eGFR and serum albumin concentrations on PK parameters of unbound teicoplanin were proposed. Importantly, our study further highlights the importance of guiding dosing through unbound drugs. It was recommended that in clinical, a reasonable dosing regimen should be designed according to the patient’s eGFR and serum albumin concentrations, which was a key step to achieve individualized dosing.

## Data Availability

The raw data supporting the conclusion of this article will be made available by the authors, without undue reservation.
