# Supplementary material for: Population pharmacokinetics and dosing optimization of unbound teicoplanin in Chinese adult patients
Source: Front Pharmacol. 2022 Nov 23;13:1045895. doi: 10.3389/fphar.2022.1045895 (PMC9728581; doi:10.3389/fphar.2022.1045895)
Supplement: Supplementary file 1 [file Table1.DOCX]

**SUPPLEMENTARY MATERIAL**

**Table 1 Simulation results for the recommended dosing regimen**

| **eGFR**  **(mL/min)** | **ALB**  **(g/L)** | **Most infection induced by Gram-positive bacteria**  **(Target unbound C_through_=0.75 mg/L)** | | | | **Endocarditis and severe infection**  **(Target unbound C_through_ = 1.13 mg/L)** | | | |
| --- | --- | --- | --- | --- | --- | --- | --- | --- | --- |
|  |  | **simulation results of loading dose** | | **simulation results of maintenance dose** | | **simulation results of loading dose** | | **simulation results of maintenance dose** | |
|  |  | **dose (mg)** | **PTA ^1^(%)** | **dose (mg)** | **PTA^2^ (%)** | **dose (mg)** | **PTA^3^ (%)** | **dose (mg)** | **PTA^4^ (%)** |
| 20 | 15 | 400 | 96.2 | 400 | 94.3 | 400 | 89.5 | 400 | 83.2 |
|  | 20 | 400 | 97.4 | 200 | 90.2 | 400 | 84.5 | 400 | 88 |
|  | 25 | 400 | 94.7 | 200 | 90.6 | 600 | 94.3 | 200 | 83.7 |
|  | 30 | 400 | 87.1 | 200 | 88.4 | 600 | 87.4 | 200 | 82.3 |
|  | 35 | 600 | 93.4 | 200 | 93.6 | 800 | 88.7 | 200 | 86.3 |
|  | 40 | 600 | 88.6 | 200 | 91.4 | 800 | 82 | 200 | 81.7 |
| 30 | 15 | 400 | 90.7 | 400 | 86.7 | 600 | 92 | 600 | 83.9 |
|  | 20 | 400 | 95.7 | 400 | 94 | 600 | 95.2 | 400 | 86.9 |
|  | 25 | 400 | 92 | 200 | 81.8 | 600 | 90.5 | 400 | 88.7 |
|  | 30 | 400 | 80.3 | 200 | 80.3 | 600 | 83.4 | 400 | 86.3 |
|  | 35 | 600 | 92.3 | 200 | 90.3 | 800 | 88.6 | 400 | 90.5 |
|  | 40 | 600 | 85.8 | 200 | 85.6 | 1000 | 91.7 | 200 | 85 |
| 45 | 15 | 400 | 80.3 | 400 | 80.7 | 600 | 82.7 | 600 | 83.5 |
|  | 20 | 400 | 89.9 | 400 | 88.4 | 600 | 88.4 | 600 | 85.7 |
|  | 25 | 400 | 87.6 | 400 | 90.2 | 600 | 85.4 | 600 | 88.9 |
|  | 30 | 600 | 96 | 200 | 85.3 | 800 | 93.3 | 400 | 86.6 |
|  | 35 | 600 | 91.4 | 200 | 81.1 | 800 | 84.7 | 400 | 82.1 |
|  | 40 | 600 | 82.8 | 200 | 80.7 | 1000 | 88.7 | 400 | 87.9 |
| 60 | 15 | 600 | 86.5 | 600 | 80.1 | 800 | 82.3 | 800 | 80.2 |
|  | 20 | 400 | 82.8 | 400 | 80.7 | 600 | 81.1 | 600 | 80.1 |
|  | 25 | 400 | 80.7 | 400 | 84.1 | 800 | 93.6 | 600 | 86.7 |
|  | 30 | 600 | 95.3 | 400 | 91.5 | 800 | 90.4 | 600 | 88.6 |
|  | 35 | 600 | 89.7 | 400 | 91.2 | 800 | 85.1 | 600 | 88.5 |
|  | 40 | 600 | 83.5 | 400 | 88.2 | 1000 | 87.3 | 400 | 85 |
| 90 | 15 | 800 | 83.8 | 800 | 80.3 | 1000 | 80.8 | 1000 | 80.2 |
|  | 20 | 600 | 86.9 | 600 | 81.3 | 800 | 85.1 | 800 | 72.6 |
|  | 25 | 600 | 93.5 | 600 | 88.6 | 800 | 87.9 | 800 | 83.9 |
|  | 30 | 600 | 91 | 400 | 80.2 | 800 | 86.6 | 600 | 80.5 |
|  | 35 | 600 | 85.8 | 400 | 84 | 1000 | 91.9 | 600 | 85.6 |
|  | 40 | 800 | 91.8 | 400 | 89.8 | 1000 | 86.5 | 600 | 82.7 |
| 130 | 15 | 1000 | 80.3 | 1000 | 80.2 | 1200 | 80.8 | 1200 | 80.2 |
|  | 20 | 800 | 87.9 | 800 | 80.7 | 1000 | 81.5 | 1000 | 82.2 |
|  | 25 | 600 | 84.8 | 600 | 80.3 | 1000 | 87.9 | 800 | 83.3 |
|  | 30 | 600 | 84.8 | 600 | 84.2 | 1000 | 90.5 | 800 | 83.4 |
|  | 35 | 600 | 80.5 | 600 | 86.8 | 1000 | 87.1 | 800 | 83.1 |
|  | 40 | 800 | 90.9 | 400 | 80.8 | 1000 | 80.1 | 800 | 82.9 |

Note: ALB- albumin, C_trough_- trough concentration, eGFR- estimated glomerular filtration rate, PTA- probability of target attainment

1. The PTA for achieving a target unbound C_through_ of 0.75 mg/L at 48 h after the first dose;
2. The PTA for achieving a target unbound C_through_ of 0.75 mg/L at 96 h after the first dose;
3. The PTA for achieving a target unbound C_through_ of 1.13 mg/L at 48 h after the first dose;
4. The PTA for achieving a target unbound C_through_ of 1.13 mg/L at 96 h after the first dose.
